# Exploring Immediate Photon Effects From 635 nm Light on Mitochondrial Bioenergetics

**DOI:** 10.1002/jbio.202500162

**Published:** 2025-07-10

**Authors:** Natasha F. Mezzacappo, Natalia M. Inada, Edilene S. Siqueira‐Santos, José Dirceu Vollet‐Filho, Roger F. Castilho, Michael L. Denton, Vanderlei S. Bagnato

**Affiliations:** ^1^ São Carlos Institute of Physics University of São Paulo (USP) São Carlos SP Brazil; ^2^ School of Medical Sciences, Department of Pathology University of Campinas (UNICAMP) Campinas SP Brazil; ^3^ Air Force Research Laboratory Bioeffects Division Texas USA; ^4^ Department of Biomedical Engineering Texas A&M University Texas USA

**Keywords:** bioenergetics, laser, mitochondria, photobiomodulation, proton leak, respirometry

## Abstract

Visible light primarily targets mitochondria at the cellular level, but photon interaction mechanisms are still not fully understood. This study examined the in vitro impacts of 635 nm laser irradiation using mitochondria isolated from mouse liver. Mitochondria samples were irradiated for 330 s inside the respirometer chamber, with delivered powers ranging from 100 to 800 mW, corresponding to power densities ranging from 31.6 to 211.7 mW/cm^2^ inside the chamber. Analysis of real‐time oxygen consumption showed an elevated proton leak during ATP synthase inhibition at 800 mW (211.7 mW/cm^2^, 69.9 J/cm^2^), suggesting enhanced permeability of the mitochondrial inner membrane. Under different experimental conditions, post‐irradiation analysis revealed increased basal respiration with 400 mW (129.1 mW/cm^2^, 42.6 J/cm^2^) and 800 mW, along with increased susceptibility to Ca^2+^‐triggered mitochondrial swelling. The investigation of mitochondrial bioenergetics demonstrated that red light induces transient and localized effects, highlighting the complexities of cellular and mitochondrial photostimulation mechanisms.

## Graphical abstract

The investigation of in vitro impacts of 635 nm laser irradiation in mitochondrial bioenergetics demonstrated that red light induces transient and localized effects, highlighting the complexities of cellular and mitochondrial photostimulation mechanisms.

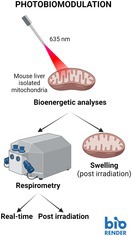

## 1 Introduction

There are many clinical effects promoted by photobiomodulation (PBM) due to its action on cellular regeneration, leading to wound healing, pain relief, and reducing inflammation [1, 2]. These therapeutic effects are typically associated with mitochondria being the main site of photon absorption in mammalian cells [2, 3, 4, 5, 6, 7, 8, 9, 10, 11, 12, 13, 14, 15, 16, 17, 18, 19, 20, 21, 22, 23, 24, 25, 26, 27]. Mitochondria are the fundamental energy centers in eukaryotic cells, producing up to 95% of cellular energy [28]. Most of the cellular energy comes from oxidative phosphorylation (OXPHOS) [28], a mechanism that produces adenosine triphosphate (ATP) molecules, which serve as fuel in numerous metabolic pathways [29]. Consequently, mitochondria are crucial for many cellular functions, including the regulation of apoptosis, calcium balance, and production of reactive oxygen species (ROS) [30].

Passarella and coworkers were the first authors to show many properties of the irradiation of isolated mitochondria at 632.8 nm, such as the changes in the optical features and the oxidative metabolism, with emphasis on the alterations in the nicotinamide adenine dinucleotide (NADH)‐linked dehydrogenase reactions, the increased ATP production, and the alterations in the mitochondrial structure [5, 6, 7]. These and other studies revealed important insights into the interaction of red light with mitochondria (see Refs. [8, 9, 10, 11]).

The prevailing hypothesis for how light (especially in the red and near infrared region) affects mitochondria centers on the absorption of photons by respiratory complex IV, also known as cytochrome c oxidase (CCO), a key component of the mitochondrial electron transport system (ETS). A pioneering study by Karu and colleagues demonstrated that exposing cells to light (across various wavelengths) enhances mitochondrial metabolism [3, 31]. This enhancement was linked to an increase in free nitric oxide (NO), suggesting that NO is released from the CCO's active site [3, 31, 32]. The NO hypothesis states that when present in high concentrations, NO inhibits CCO by directly competing with molecular oxygen (O_2_) for binding at the enzyme's active center [2]. Since cells that are damaged tend to have harmful levels of NO, reversing this inhibition of CCO would consequently stimulate and restore normal ATP production, thereby accounting for the positive clinical outcomes observed [2].

Amaroli and colleagues explored different approaches using near‐infrared light. They observed that when mitochondria were irradiated at 808 nm, there was an increase in oxygen consumption [12]. This increase was linked to enhanced activity of respiratory complexes III–IV and ATP synthase, while complexes II–III remained unaffected [12]. They hypothesized that 808 nm light might interact with the heme groups or iron–sulfur proteins within complexes III and IV [12]. Other research has shown that 1064 nm irradiation also boosts the activity of complexes I, III, IV, and ATP synthase. This suggests interactions with Fe–S proteins, Cu‐centers, and water molecules [13].

Another frequently observed mechanism in in vitro experiments involving irradiated mitochondria is the increase in ROS, such as hydroxyl radicals, superoxide, singlet oxygen, and hydrogen peroxide [33]. Although excessive accumulation of ROS can cause mitochondrial and cellular damage [30], studies have shown that a moderate increase in ROS triggered by light exposure can benefit cells under certain conditions [1, 2, 33, 34]. At low concentrations, ROS play a role in various cellular signaling pathways [35]. In addition to normalizing ATP levels and regulating proteins associated with cell proliferation and differentiation, photon‐induced ROS production is believed to activate transcription factors such as NF‐κB, which are crucial for regulating gene expression, immune responses, stress responses, and apoptosis [1, 33]. However, the effects of light‐induced ROS can be either beneficial or harmful [2]. The varying responses observed across different studies can be attributed to differences in light parameters, such as power densities and wavelength [2].

Research in PBM typically emphasizes the positive, modulatory effects of light irradiation, such as improved cell proliferation and metabolism. However, these outcomes are generally observed hours or even days after irradiation [18, 36, 37]. Although the effects of PBM have been explored across various samples, utilizing different light sources, wavelengths, and analysis methods [4, 5, 6, 19, 21, 22, 23, 24, 31, 32, 38] several potential mechanisms involved in photon interactions with mitochondria still remain not fully understood [1, 2]. Recent bioenergetics studies in the field primarily focus on analyzing ETS enzymes and ATPase, as well as the impact of NO levels, often irradiating samples prior to analysis [19, 39]. However, this approach provides limited insight into photon interactions in real‐time. To our knowledge, Pope et al. conducted the first study aiming at the real‐time evaluation of light on mitochondria [36]. They measured real‐time respirometry in mitochondria isolated from the porcine heart, exposing them to low‐level irradiation (2.5 mW) at 808 nm [36]. However, they did not observe any significant changes in the measured parameters using the selected respirometry protocol [36].

The present study assesses the effects of in vitro PBM on mitochondrial respiration, both in real‐time and immediately following 635 nm irradiation. We selected the in vitro model of isolated mitochondria, which offers the ability to study fundamental processes with high metabolic fidelity, free from the influence of cellular machinery, while preserving the mitochondrion's intrinsic functions [37, 40]. This approach provides a valuable biochemical method for exploring the organelle's mechanisms and the effects that occur during or shortly after irradiation [37, 40]. For irradiation, a 635 nm continuous wave (CW) diode laser was chosen, given its well‐established efficacy in PBM, demonstrated in both clinical and in vitro studies, with proven anti‐inflammatory and regenerative effects [41]. Furthermore, red light, due to its higher photon energy, is more effective than NIR in driving electrochemical tissue changes [34]. For the analysis, we questioned what potential immediate effects of irradiation could be measured, and which experimental design would be capable of measuring such effects. High‐resolution respirometry (HRR) is the most suitable technique to evaluate the respiratory chain and OXPHOS in isolated mitochondria, measuring responses in real‐time through oxygen consumption, unlike other mitochondrial techniques as direct measurements of membrane potential that require fluorescent probes, which could interfere with the analysis [42, 43]. Therefore, we chose to use HRR for both real‐time and post‐irradiation analysis, as well as to assess mitochondrial swelling by spectrophotometry [44, 45, 46, 47], to correlate the bioenergetics results with an evaluation of mitochondrial permeability transition pore (mPTP) opening [48, 49, 50].

## 2 Experimental

### 2.1 Reagents

Adenosine diphosphate (ADP, #A5285), bovine serum albumin (BSA, #A9418), butylated hydroxytoluene (BHT, #B1378), cyclosporin A (CsA, #30024), cytochrome C (#C7752), CCCP (#C2759), dibasic potassium phosphate (K_2_HPO_4_, #P5504), EGTA (#E4378), glutamic acid (#G1501), HEPES (#H3375), malic acid (#M1000), and oligomycin A (#O4876) were purchased from Sigma–Aldrich (St. Louis, MO, USA). Sucrose, potassium chloride (KCl), and magnesium chloride (MgCl_2_) were obtained from Synth (Diadema, São Paulo, Brazil). Calcium chloride (CaCl_2_) and potassium hydroxide (KOH) were obtained from Êxodo Cientifica (Sumaré, São Paulo, Brazil). All solutions were prepared using ultrapure Milli‐Q water. The pH of all solutions was adjusted to 7.2 with KOH.

### 2.2 Animals

Wild‐type C57BL/6 adult female mice were obtained from the Animal Facility of Ribeirão Preto Campus of the University of São Paulo (USP‐RP). The animals were housed in ventilated rack systems (Alesco, Brazil) with individual air supply to the cages, maintained under a 12 h light/dark cycle. Mice had free access to water and were fed *ad libitum*. The animals were acclimated, and experiments were conducted on mice aged between 10 and 24 weeks old. All procedures were conducted in compliance with the National Council for the Control of Animal Experimentation (CONCEA) guidelines and were approved by the Ethics Committee on the Use of Animals of the São Carlos Institute of Physics (CEUA/IFSC No. 3620051018).

### 2.3 Isolation of Mice Liver Mitochondria

The technique of differential centrifugation was employed to isolate mitochondria from the liver [51], conducting the procedures always at 4°C or in an ice bath. The mouse euthanasia was performed through cervical dislocation without anesthesia to avoid alterations in liver metabolism, as shown in the literature [37, 52, 53, 54, 55, 56]. Death was confirmed by checking the lack of vital signs. The liver was quickly excised through a peritoneal incision and immediately placed into a 50 mL conical plastic tube containing ice‐cold isolation buffer I (250 mM sucrose, 0.5 mM EGTA, and 10 mM HEPES; pH 7.2). It was then minced with scissors in an ice bath and homogenized in a Potter–Elvehjem tissue homogenizer (NT136, Novatecnica, Brazil) at 300 rpm, using a glass tube and a polytetrafluoroethylene (PTFE) pistol. The homogenate was then transferred to a 50 mL conical plastic tube, with the volume completed to 40 mL with isolation buffer 1, and centrifuged using a benchtop refrigerated centrifuge (Allegra X‐30R, fixed angle rotor C0650, Beckman Coulter, USA) for 10 min at 800 × *g* and 4°C. The obtained supernatant was transferred to a new conical 50 mL plastic tube and centrifuged at 7700 × *g* and 4°C for 10 min. After discarding the supernatant, the resulting pellet was gently brushed with a pony hair round brush (size 0), avoiding any residual blood cell at the bottom of the pellet, then transferred to a new 50 mL conical plastic tube with 25 mL of isolation buffer II (250 mM sucrose, 0.3 mM EGTA, and 10 mM HEPES; pH 7.2) and centrifuged again for 10 min at 7700 × *g* and 4°C. The final mitochondrial pellet was washed three times with isolation buffer III (250 mM sucrose and 10 mM HEPES; pH 7.2) and resuspended in an aliquot of the same buffer. The Bradford method [57] was used to quantify the protein concentration in the mitochondria sample, using different standard BSA concentrations.

### 2.4 Oxygen Consumption Measurements

The Oxygraph‐2k respirometer (OROBOROS, Austria) was used to monitor the mitochondrial oxygen consumption through the HRR technique. Air calibration was performed daily before the experiments at 28°C, with a stirrer speed of 750 rpm in the 2 mL chambers of the Oxygraph‐2k, using the standard reaction buffer defined in [51]: 125 mM sucrose, 65 mM KCl, 2 mM K_2_HPO_4_, 1 mM MgCl_2_, and 10 mM HEPES (pH 7.2). The oxygen flux (negative time derivative of oxygen concentration) was corrected for instrumental background [58].

**FIGURE 1:**
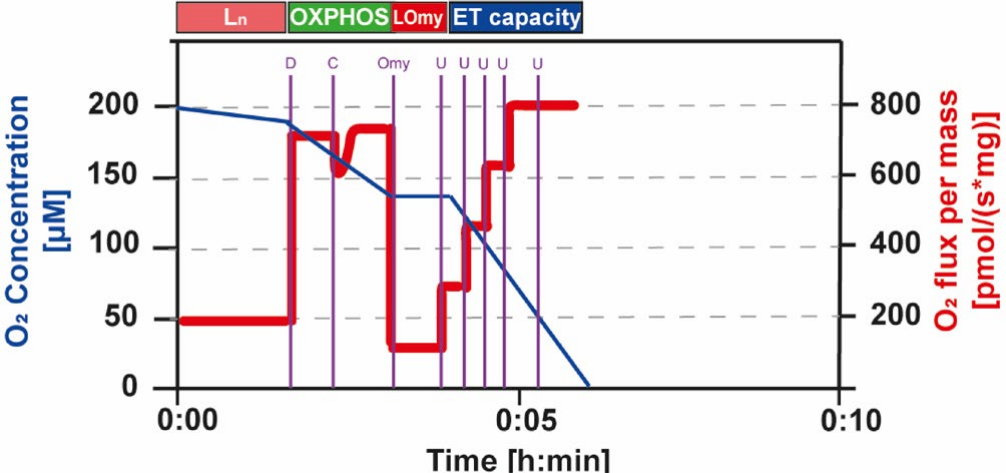
Representative trace for the SUIT protocol applied in the OCR analysis, based on Gnaiger representations [59]. Vertical purple lines mark the titrations: D: 500 μM ADP; C: 10 μM cytochrome C; Omy: 1 μg/mL oligomycin; U: uncoupler (50 nM CCCP each titration).

Isolated liver mitochondria samples (0.5 mg/mL) were incubated in the respirometer chambers in the standard reaction buffer with complex I substrates (5 mM glutamate and 2.5 mM malate). The oxygen consumption rates (OCR) were measured by the following SUIT (substrate–uncoupler–inhibitor titration) protocol (Figure 1), adapted from SUIT‐006 [59]: 500 μM ADP, 10 μM cytochrome C, 1 μg/mL oligomycin, and 50–250 nM CCCP stepwise titrations. Subsequently, the parameters listed in Table 1 were determined based on these titrations.

**TABLE 1:** Bioenergetic parameters associated with the analysis of oxygen consumption (adapted from [59]).

Parameter	Occurrence	Meaning
LEAK_*n*_ (*L*_*n*_)	Mitochondria with substrates	Mitochondrial respiration without exogenous ADP
OXPHOS (*P*)	ADP addition	Oxidative phosphorylation
LEAK_Omy_ (*L*_Omy_)	Oligomycin addition	Proton leak stage, when respiration is measured after exhausting OXPHOS or after inhibition of ATP synthase by oligomycin
ET capacity (*E*)	CCCP addition	Maximal respiratory capacity of ETS after uncoupling the respiration
Respiratory control ratio (RCR)	*P*/*L*_Omy_	Indicator of the mitochondria sample quality, evaluating the defects in the ETS, ATP synthase, and the variation in proton leak [43] “Healthy” and high‐quality samples will have high respiratory control [60]
Cyt C control efficiency (*J*_cyt_)	Cyt C addition *J*_cyt_ = (*P*_cyt–_ *P*)/*P*_cyt_	Indicator of mitochondrial outer membrane integrity, since damages to the membrane result in Cyt C release [59]
*E* − *L* coupling efficiency (*J*_*E*−*L*_)	*J*_*E*−*L*Omy_ = (*E* − *L*_Omy_)/*E*	Indicator of ETS preservation and coupling to ADP phosphorylation [59]
*E* − *P* control efficiency (*J*_*E*−*P*_)	*J*_*E*−*P*_ = (*E* − P)/*E*	Indicator of the OXPHOS capacity limitation due to the phosphorylation system capacity [59]

### 2.5 Irradiation Parameters

#### 2.5.1 Real‐Time Analysis

**FIGURE 2:**
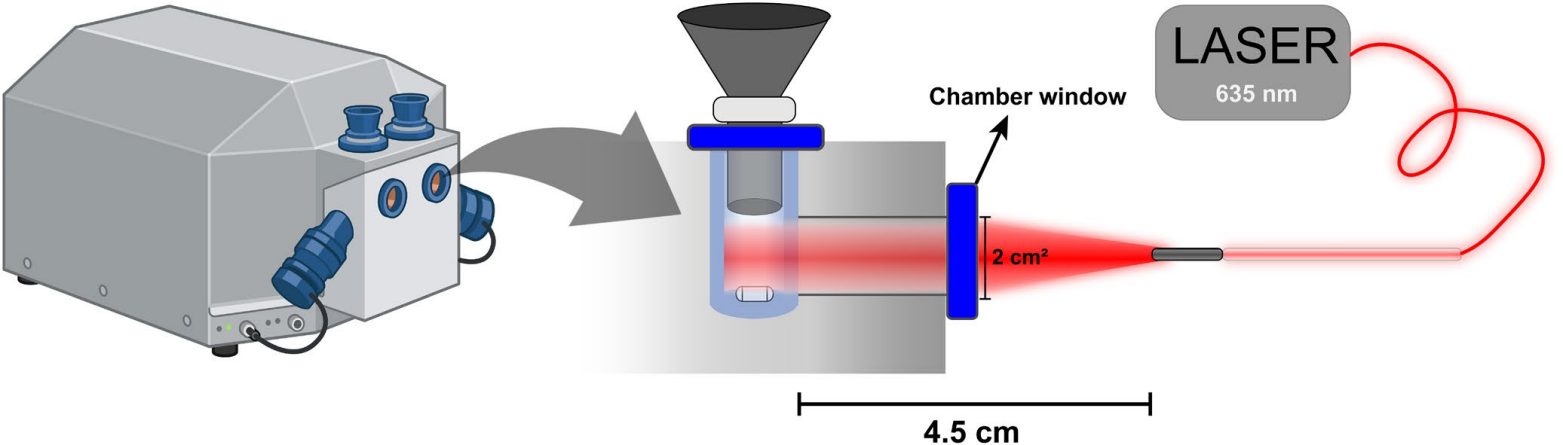
Experimental configuration for irradiating the sample externally with 635 nm diode LASER delivered with a uniform distribution optical fiber. This image was modified from [61] and was made partially with BioRender.

Sample irradiation in real‐time was conducted as described in [29] with modifications. Irradiation was applied externally while oxygen consumption was measured for about 330 s, with the chamber closed, to evaluate the immediate effects of light in real‐time (Figure 2). The stirrer's speed was set at 750 rpm to homogenize the mitochondrial solution and ensure uniform irradiation of the organelles. The internal illumination of the chambers was switched off during the entire time of irradiation and measurements and in the dark controls.

The external light exposure system consisted of a 635 nm CW diode laser (Ceramoptec, GmbH, Germany) with a uniform distribution optical fiber (Frontal Light Distributor, FD1, Medlight SA, Ecublens, Switzerland) to deliver a uniform circular laser beam spot (Figure 2). The light output was positioned 4.5 cm from the Duran glass cylindrical chamber, which was placed inside the respirometer, to create a beam that most closely approximates the diameter when it reaches the chamber window. The FD1 device generates a beam with a divergence angle of 34.7°, with ±15% uniformity. The beam spot area was large enough to cover the sensor area of the power/energy meter (LabMax TOP, COHERENT, USA, sensor PM10 model), which was 2.89 cm^2^. This was the spot area considered to calculate the average delivered power densities. These and other irradiation parameters are presented in Table 2. Spectra from outside (at a 4.5 cm distance) and inside the chamber were measured using an isotropic probe optical fiber (IP85 model, Medlight SA, Switzerland) connected to a USB4000 fiber optic spectrometer (OceanOptics, EUA) (Figure S1A), with the SpectraSuite (OceanOptics, EUA) software. The power/energy meter was used to measure the laser power outside the respirometer (Figure S1B). Power densities calculated from the measurements outside (Section S1) were correlated with the area under the corresponding spectra curves, generating a calibration curve (Figure S2). This calibration curve was used to measure the power densities inside the chamber, using the respective spectra. Irradiation characterization details are described in Section S1. The oxygen consumption analysis followed the protocol described in Figure 1.

**TABLE 2:** Parameters related to the 635 nm laser irradiation considering the experimental setup.

Delivered power (mW)	100	200	400	800
Irradiation time (s)	330	330	330	330
Average delivered power density (mW/cm^2^)	34.6	69.2	138.4	276.8
Delivered fluence (J/cm^2^)	11.4	22.8	45.7	91.3
Delivered energy (J)	33	66	132	264
Average power density inside the chamber (mW/cm^2^)	31.6 ± 0.4	63.7 ± 0.9	129.1 ± 1.1	211.7 ± 1.0
Fluence inside the chamber (J/cm^2^)	10.4 ± 0.1	21.0 ± 0.3	42.6 ± 0.4	69.9 ± 0.3

Temperatures for each irradiation condition are shown in Table 3. The temperature inside the closed chambers was measured with a needle thermocouple (MT‐29/1HT Needle Microprobe, Physitemp Instruments). Although the respirometer chambers are integrated with an electronic Peltier system that controls the temperature with high precision (2°C–45°C, ± *0.001°C [58]), there were slight increases in the temperature of the solution at the highest laser power intensities during real‐time oxygen consumption measurements, as previously reported for the same setup [61]. Hence, the temperature of the Peltier system in the respirometer (block temperature) was modified accordingly (Table 3) to ensure the desired stable temperature of the solution was maintained during the measurements and to minimize thermal effects.

**TABLE 3:** Temperature parameters of real‐time analysis.

Power (mW)	Block temperature (°C)	Final temperature (°C)
0	28	28.0 ± 0.1
100	28	28.0 ± 0.1
200	28	28.0 ± 0.1
400	27.5	28.0 ± 0.1
800	27	28.0 ± 0.1

#### 2.5.2 Post‐Irradiation Analysis

The experimental setup (Figure 2) was used for irradiation, with modifications: the chamber was kept open during the irradiation period (330 s) to prevent potential oxygen depletion caused by a closed chamber from affecting the conditions before measurements, and the block temperature was maintained at 28.0°C ± 0.1°C. Once irradiation was complete, the chamber was immediately closed, and oxygen consumption was measured using the protocol described in Figure 1. For mitochondrial swelling, the sample was collected immediately after irradiation, and the protocol for swelling analysis was performed as described in Section 2.6. Table 2 provides the details of the irradiation parameters.

### 2.6 Mitochondrial Swelling

When mitochondrial swelling occurs, there are changes in the organelle's refractive index due to a reduction in light scattering, which can be observed spectrophotometrically [44, 45, 46, 47, 48, 49, 50]. Mitochondrial swelling was measured using a microplate spectrophotometer reader (Multiskan GO, Thermo Scientific, USA) in kinetics mode with 96‐well plates, according to the protocol described by Parks et al. [48]. Following irradiation, aliquots from the sample (at a concentration of 0.5 mg/mL in standard reaction buffer) were added to the wells, and light transmission was measured at 520 nm over 15 min. At the 1‐min mark, 50 μM of CaCl_2_ was added to select wells to induce swelling, and measurements were taken for the remaining 15 min. For each sample, two control measurements were carried out: one with only the medium and mitochondria, and another containing 1 μM of CsA, in which 50 μM of CaCl_2_ was added to the mitochondria at the 1‐min mark. CsA suppresses the membrane permeability transition [48, 49, 50], thus preventing mitochondrial swelling [62]. Additionally, swelling measurements were conducted on samples taken directly from the stock without any procedures applied (initial control).

### 2.7 Data Analysis

OCR data were recorded as O_2_ flux normalized per mass (pmol/(s*mg)) by DatLab 7.4 (OROBOROS, Austria) and analyzed through Origin 2022 (OriginLab, USA). All measurements are presented as mean values from at least three separate experiments, with the corresponding standard deviations. The Shapiro–Wilk test was used to assess the normality of all groups. One‐way ANOVA was applied to compare groups. Results were considered statistically significant when *p*‐value < 0.05.

Light scattering data from mitochondrial swelling were acquired as arbitrary units by SkanIt Software 7.0.2 RE (Thermo Scientific, USA) and analyzed using Origin 2022 software (OriginLab, USA). Blank spectra were subtracted from the light scattering spectra, which were normalized (from 0 to 1) concerning the maximum value. All measurements are reported as mean spectra of duplicates from at least three different experiments with corresponding standard deviations. Data were quantified by calculating the variation of each normalized light scattering (Δ*L*.*S*.) between 100 and 400 s and are reported as mean values with the corresponding standard deviations. One‐way ANOVA was applied to compare groups. Results were considered significantly different with a *p*‐value < 0.05.

## 3 Results

### 3.1 Real‐Time Analyses of Mitochondrial Respiration

**FIGURE 3:**
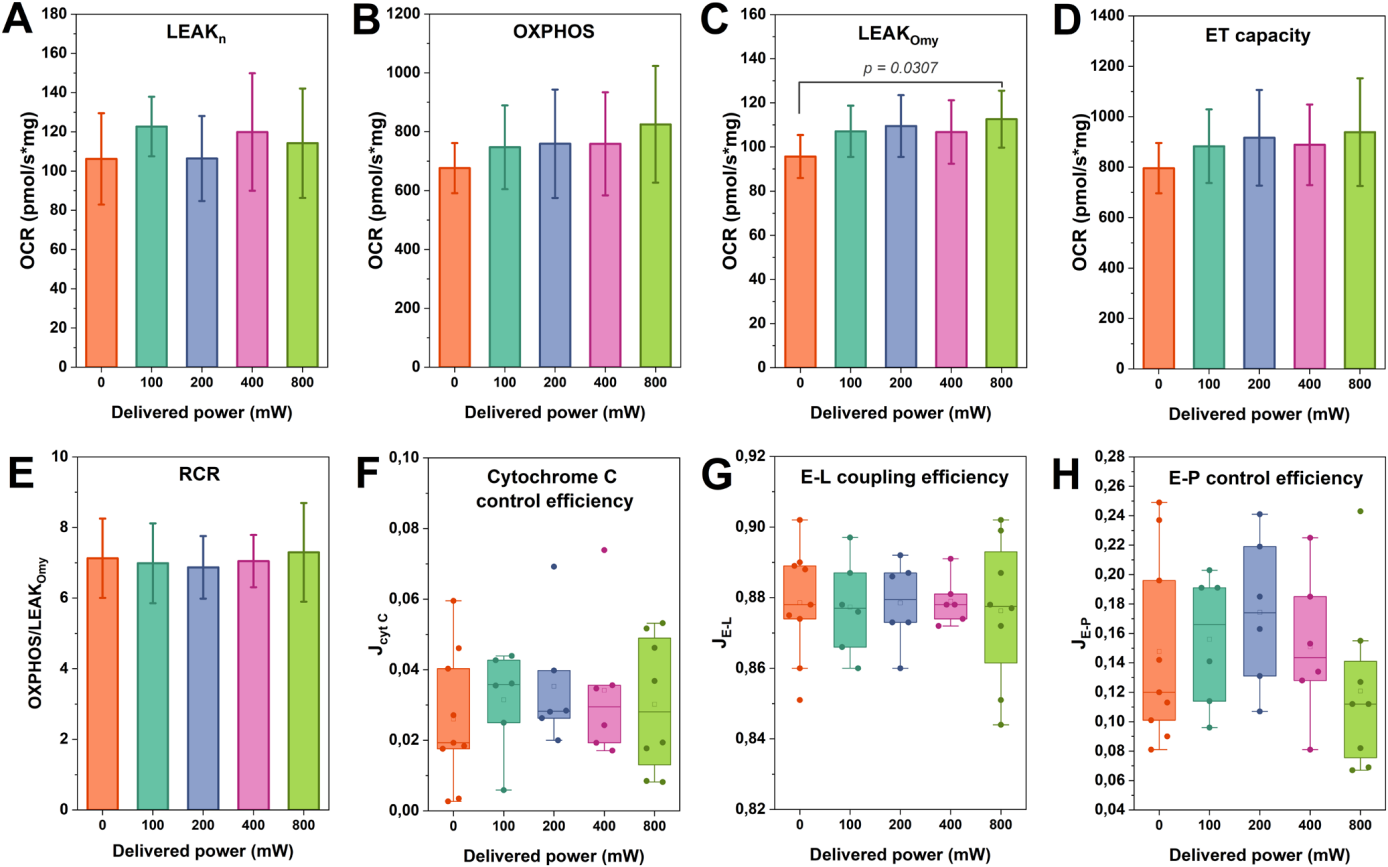
Oxygen consumption parameters for real‐time irradiation. The details of each bioenergetic parameter are provided in Table 1, and the irradiation parameters are provided in Table 2. One‐way ANOVA (*N* = 5 animals) was used to compare the irradiated groups with the control (0 mW). Data are shown as mean values (*N* = 6–9 measurements), standard deviations, and *p* < 0.05.

The OCR obtained from the real‐time analyses and the corresponding calculated parameters are shown in Figure 3. A 17.7% increase in OCR was observed in the LEAK_Omy_ when the 800 mW (211.7 mW/cm^2^, 69.9 J/cm^2^) irradiation was compared to the dark control (0 mW), suggesting an enhancement in proton leak when ATP synthase was inhibited by oligomycin. No significant differences were observed in the other OCR parameters for any of the remaining irradiation conditions. Cytochrome C efficiency remained unchanged, suggesting that despite the increase in proton leak, no apparent damage occurred to the mitochondrial outer membrane when compared to the dark control.

### 3.2 Post‐Irradiation Analyses of Mitochondrial Respiration

**FIGURE 4:**
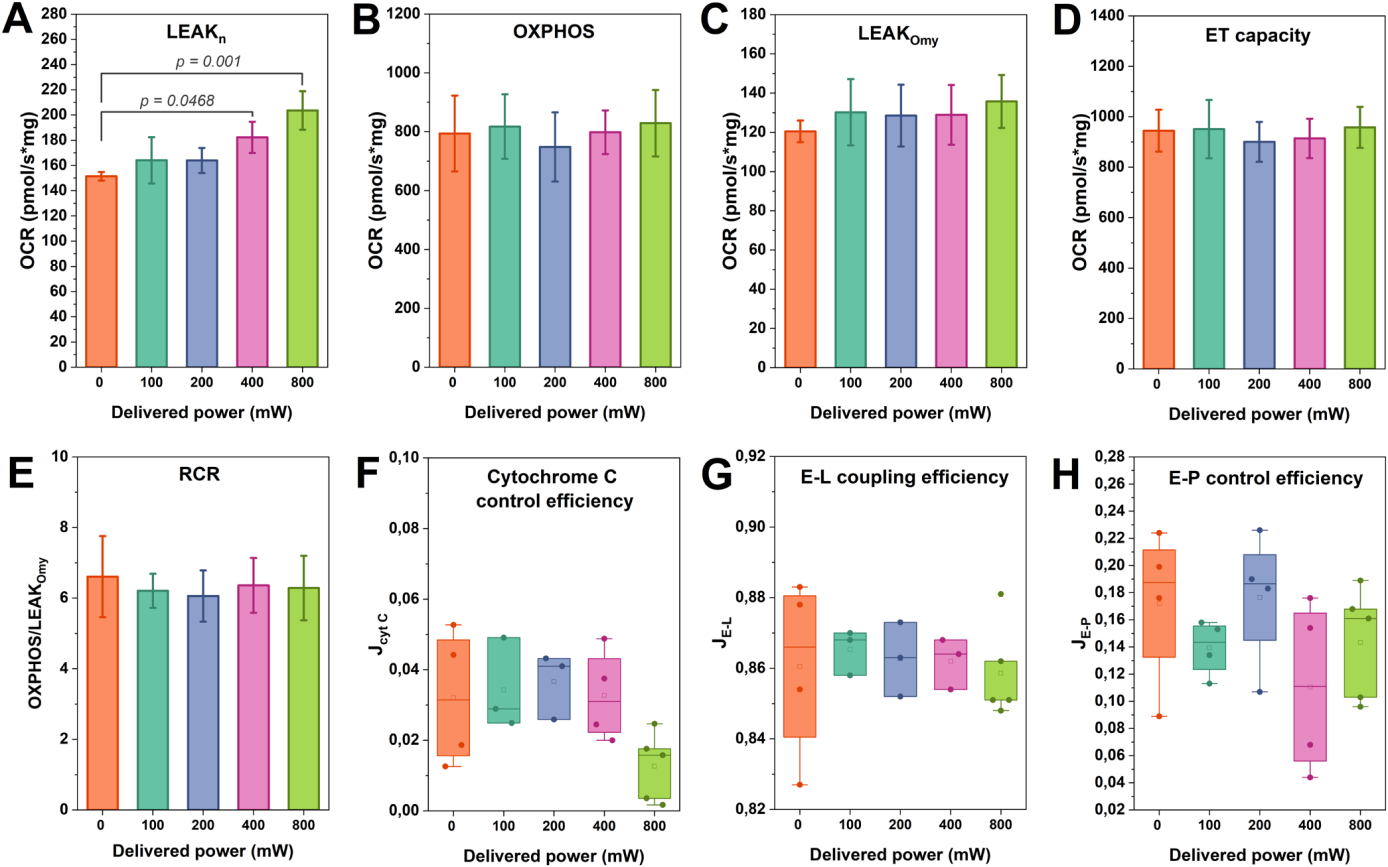
Oxygen consumption parameters post‐irradiation. The details of each parameter are provided in Table 1, and the irradiation parameters are provided in Table 2. One‐way ANOVA (*N* = 5 animals) was used to compare the irradiated groups with the control (0 mW). Data are shown as mean values (*N* = 3–4 measurements), standard deviations, and *p* < 0.05.

Figure 4 shows the OCR parameters recorded immediately following irradiation. This analysis is crucial as some effects occurring during irradiation may persist afterward. The LEAK_*n*_ state showed notable increases at the highest fluences, with a 20.4% increase at 400 mW (129.1 mW/cm^2^, 42.6 J/cm^2^) and a 34.6% increase at 800 mW (211.7 mW/cm^2^, 69.9 J/cm^2^). No significant changes were observed in the other irradiation conditions and parameters analyzed, including cytochrome C efficiency, which suggests that the mitochondrial outer membrane was not damaged.

### 3.3 Mitochondrial Swelling Post‐Irradiation Analyses

**FIGURE 5:**
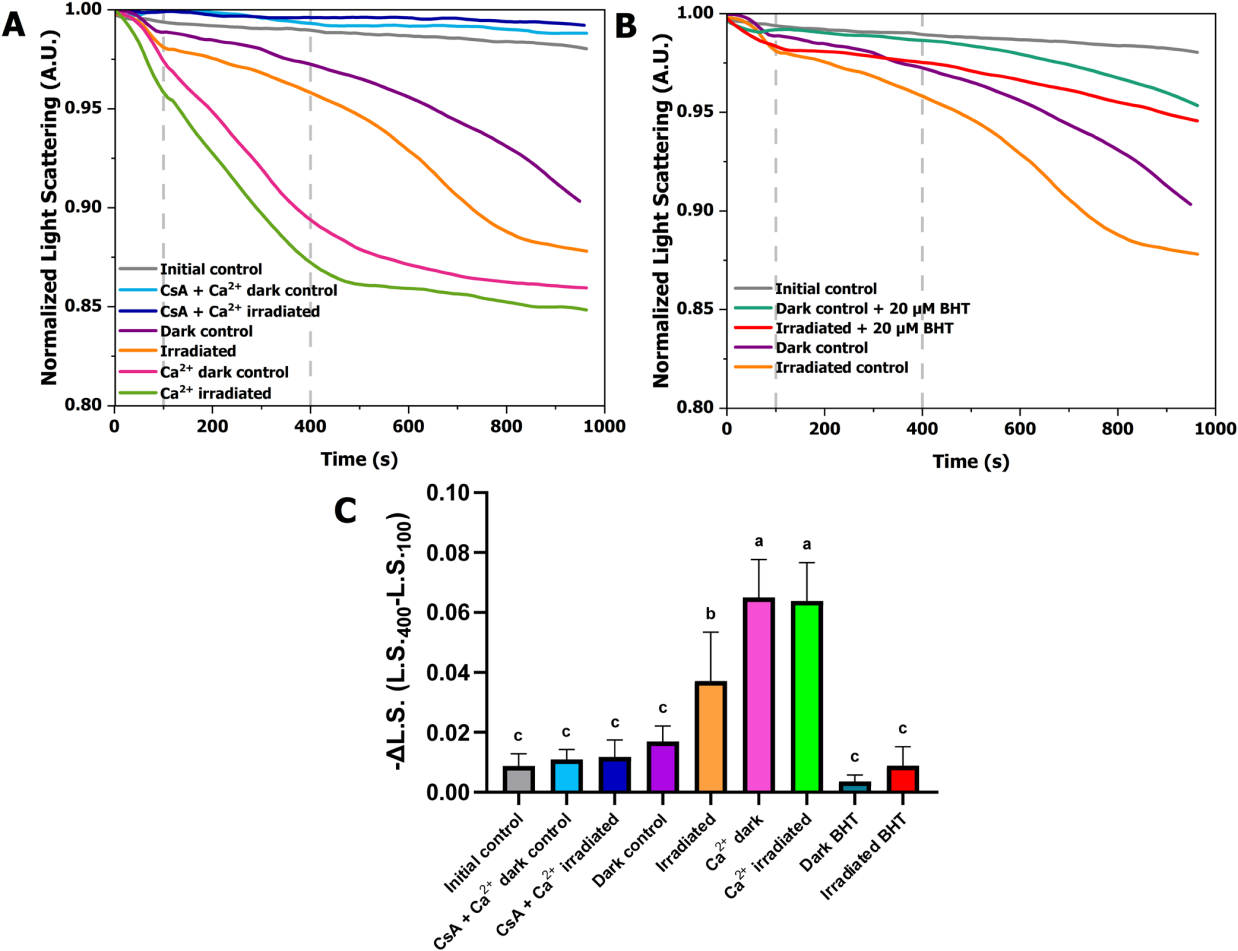
Mitochondrial swelling was assessed by monitoring light scattering at 520 nm over time. (A) Swelling was compared with and without CsA and added Ca^2+^, between the dark control and irradiated groups (800 mW, 211.7 mW/cm^2^, 69.9 J/cm^2^). Gray trace: no incubation or Ca^2+^ addition; dark blue trace: samples treated with CsA and Ca^2+^ following dark incubation; light blue trace: samples treated with CsA and Ca^2+^ following the exposure to irradiation; purple trace: samples incubated in the dark without any treatment; orange trace: samples exposed to irradiation; pink trace: samples treated with Ca^2+^ following dark incubation; green trace: samples treated with Ca^2+^ following the exposure to irradiation. (B) Swelling was compared with and without 20 μM BHT. Gray, purple, and orange traces represent the same conditions as in (A). Dark green trace: samples incubated in the dark with BHT; red trace: sample exposed to irradiation in the presence of BHT. Vertical reference lines in (A) and (B) mark the 100 and 400 s intervals, indicating the light scattering range (Δ*L*.*S*.) used for calculation in (C). (C) Data quantification was performed using the variation in normalized light scattering (Δ*L*.*S*.) between 100 and 400 s [63]. The values are shown as mean ± standard deviation (*N* = 4–11 measurements). Statistical significance (one‐way ANOVA; *p* ≤ 0.05) is indicated by the letters (a, b, and c) assigned to the groups, where groups with identical letters do not differ significantly.

Light scattering was used to assess the mitochondrial swelling profiles under the specified conditions, with a reduction observed at 520 nm due to organelle swelling, as shown in Figure 5. The figure presents a quantitative comparison of swelling with and without CsA, Ca^2+^ addition (Figure 5A,C), and BHT (Figure 5B,C). Based on the conditions that demonstrated significant differences in the post‐irradiation OCR analysis, irradiation at 800 mW (211.7 mW/cm^2^, 69.9 J/cm^2^) was applied to the sample. Minimal swelling was observed in the control condition (gray trace, Figure 5A), where neither incubation nor Ca^2+^ was added, a result similar to the inhibition of swelling by CsA (dark and light blue traces). Both the dark control (purple trace) and the irradiated group (orange trace) show increased swelling, with the irradiated sample showing greater and faster swelling compared to the sample kept in the dark. Furthermore, it was indicated by the quantification analysis in Figure 5C that the swelling in the dark control did not differ significantly from the swelling observed in the other control groups. When compared to the dark control, the swelling in the irradiated group was notably higher, reaching 119.5%, as shown in Figure 5C. Adding Ca^2+^ to the samples resulted in a slight increase in swelling in the irradiated group (green trace) compared to the dark control (pink trace), but no significant quantitative differences were found between the two groups (Figure 5C). The swelling observed in the dark control suggests that mitochondria become more permeable even without irradiation, with greater swelling observed in the irradiated samples. Some damage likely occurred during sample incubation, even in the dark, because the reaction medium contains trace amounts of Ca^2+^, from the ultrapure water and reagents [64]. Moreover, the addition of CsA to the samples with high Ca^2+^ resulted in a 68.2% inhibition of swelling (Figure 5C), suggesting that the opening of the mitochondrial permeability transition pore (mPTP) triggered the induction of membrane permeability [62].

The swelling profile was further evaluated in the presence of 20 μM of BHT during incubation and irradiation (Figure 5B) to assess whether oxidative stress could be responsible for the Ca^2+^‐induced opening of the mPTP. As an antioxidant, BHT protects against lipid peroxidation and ROS accumulation [65] thereby mitigating the mitochondrial swelling associated with these damages [66]. When BHT is present, there is a reduction in the swelling profile in both BHT groups (Figure 5B). Additionally, the swelling in the irradiated BHT group (76.1%) is more pronounced than in the dark BHT group (Figure 5C). These results provide strong evidence that lipid peroxidation occurs in both conditions [65], potentially linked to an elevated ROS production as a result of irradiation.

## 4 Discussion

Real‐time measurements revealed an elevation in proton leak (LEAK_Omy_) following irradiation. Proton leak, linked to respiration coupling, is a fundamental process where some protons move into the mitochondrial matrix apart from ATP synthesis [67]. Healthy mitochondria exhibit proton leak even within their native state (e.g., intact cells), suggesting that the leakage is likely driven by the activity of other enzymes in the mitochondrial inner membrane [35, 67, 68]. ATP synthase inhibited by oligomycin enables the evaluation of the OCR not linked to ATP synthesis [69, 70]. Thus, the greater proton leak observed in LEAK_Omy_ suggests a moderate uncoupling [67] and possible changes in the inner mitochondrial membrane. Simultaneously, the irradiation intensity (211.7 mW/cm^2^) and the experimental approach (continuous irradiation and stirring) may influence the activity of inner membrane carrier proteins and other mechanisms involved in mitochondrial respiration, which were not explored in this study. A potential mechanism could involve the adenine nucleotide translocase (ANT), which mediates the transport of ADP and ATP across the mitochondrial inner membrane and contributes to proton leak [71]. This was suggested by Passarella and colleagues, who demonstrated that the activation of the ADP/ATP exchange mechanism was a crucial factor driving the increase in ATP synthesis upon He‐Ne laser (632.8 nm) irradiation [7]. The authors were the first to show irradiation‐induced alterations in the characteristics of mitochondria isolated from rat liver, such as modifications in optical properties and metabolic activity linked to NADH dehydrogenase [5]. They also proposed that the increase in proton motive force induced by irradiation led to greater ATP generation [6], agreeing with their earlier hypothesis that light is absorbed by a photoacceptor located within the mitochondrial compartments [5].

Interesting, an increase in the LEAK_*n*_ was observed in the post‐irradiation measurements at irradiations of 400 mW (129.1 mW/cm^2^, 42.6 J/cm^2^) and 800 mW (211.7 mW/cm^2^, 69.9 J/cm^2^), while the LEAK_Omy_ showed no changes, which differed from the findings in the real‐time measurements. The most likely explanation is related to the variations in the total radiant exposure of light that the sample received before the LEAK_*n*_ was measured after irradiation. While the exact radiant exposure at the time of the LEAK_*n*_ measurement for the real‐time analysis cannot be determined, it is known that the sample was irradiated for a shorter duration. Since the LEAK_*n*_ state is measured within a brief interval during the irradiation, only a fraction of the total fluence was delivered at that moment. Additionally, a restoration of the proton leakage may have occurred by the time of assessment in the post‐irradiation analysis, suggesting that the alteration in the inner mitochondrial membrane could be reversible and transient. Furthermore, the enhancement in the activity of adenosine triphosphatases (or ATPases) could explain the elevated LEAK_*n*_ respiration. In this scenario, the hydrolysis of endogenous ATP into ADP may be occurring, which could slightly boost oxidative phosphorylation during basal respiration [72, 73]. Besides, the literature suggests that proton leak enhancement may be linked to higher ROS generation, acting as a protective feedback mechanism against oxidative damage. Through this mechanism, proton leakage is triggered by the increase in ROS, which reduces ROS generation and limits damage [35, 74]. Given that it is well‐established that irradiation increases ROS generation in mitochondria [1, 2, 33], this mechanism aligns with our findings, although ROS were not measured directly in our study.

The swelling analysis conducted after irradiation suggests that the opening of the mPTP (at 800 mW, 211.7 mW/cm^2^, 69.9 J/cm^2^) induced membrane permeability, and swelling was reduced in the presence of BHT, indicating that lipid peroxidation may have occurred [65]. However, one of the limitations of our study was the evaluation of mitochondrial swelling only by light scattering in mitochondrial suspensions, without direct assessment of this phenomenon by electron microscopy.

Low‐level irradiation is typically used in clinical PBM studies with red and NIR wavelengths [1, 2, 75], using either CW or pulsed light, characterized by power densities of 1–5 W/cm^2^, laser beam fluence ranging from 0.04 to 50 J/cm^2^ [76] and output power limited to less than 500 mW to prevent excessive heating [77]. Although lower power densities and fluences are generally associated with improved effects in most in vitro cell studies [24, 78, 79] due to the biphasic dose–response [77], our experimental design did not follow this pattern, as the lower irradiation parameters—from 100 mW (31.6 mW/cm^2^, 10.5 J/cm^2^) to 200 mW (63.7 mW/cm^2^, 21.1 J/cm^2^)—had no effect. As can be observed, this study applied delivered powers and fluences that were outside the specified ranges. At power levels above 400 mW, thermal effects were observed, as expected [80], which were corrected by adjusting the temperature during the real‐time analysis. However, the amount of power delivered to our sample was reduced, as the irradiation passed through the Duran glass chamber, which acted as a barrier between the laser beam and the sample, allowing only a fraction of the light source's output power to be transmitted to the sample. As a result, the total power delivered is only meaningful when the frontal area of the irradiated sample is considered, along with the volume, which includes factors such as light coupling to the glass surface, light transmission, and the cylindrical geometry of the glass chamber. These aspects are effectively represented by the power density parameter, which was quantified inside the chamber using an isotropic probe optical fiber (Section S1). In addition, a critical consideration in our experimental design is the irradiance homogeneity within the chamber. The FD1 optical fiber device employed for irradiation exhibits a beam uniformity of ±15%. Although the mitochondrial suspension was subjected to continuous agitation during the irradiation period, it is improbable that each mitochondrion received an equal photon fluence. Consequently, the observed data reflect a population‐level effect, precluding definitive conclusions regarding energy absorption at the individual mitochondrial level. This represents an inherent limitation of the current experimental design.

Moreover, a direct comparison between these experimental parameters and clinical results, in vivo models, or cell studies is not straightforward. While this study offers valuable insights using isolated mitochondria, which were chosen for their highly controlled environment, it inherently lacks the complexity of intact cellular systems. Consequently, experimental parameters must be carefully adjusted to reflect the specific limitations and conditions of each model. As stated by Felician et al., variations in outcomes often occur depending on the nature of the experiment conducted, even when the same set of parameters is used [9]. Amaroli and colleagues also highlighted the need for caution when examining light‐related phenomena in isolated mitochondria [18, 81]. This underscores one of the challenges in comparing outcomes across different PBM studies. Reaching a consensus on specific PBM effects can be challenging due to the wide variation in parameters and sample types used across studies [2, 18], and in some cases, critical details are even omitted from reports [80, 81]. Furthermore, the validity of the “low‐power paradigm” has been brought into doubt by recent in vitro studies using isolated mitochondria, which demonstrated more pronounced effects at higher power levels [19, 33, 82], suggesting that the concept of using low power may not be applicable in the context of isolated mitochondria. The observed effects in whole cells may not be directly connected to the mitochondrial pathways studied in isolated mitochondria, as demonstrated in the literature, potentially explaining this difference [20, 39, 83]. Amaroli et al. demonstrated that there is a threshold for power and exposure time beyond which certain effects become positive, at least for 810 nm [18].

Another important limitation of this study is using only a specific wavelength (635 nm) for irradiation, which limits broader conclusions about wavelength‐dependent effects. PBM responses are known to be highly wavelength‐specific [84], with different wavelengths penetrating biological tissues to varying extents and interacting with distinct chromophores. A limited number of authors in the literature have investigated the impact of various wavelengths. Most studies focus on the red [5, 6, 7, 8, 9, 10, 11, 21, 23, 24, 26, 27, 39, 83] and NIR wavelengths, as Amaroli and coworkers with 808 nm [12, 15], 810 nm [18], 980 nm [16], and 1064 nm [13]. Pope et al. used visible and NIR wavelengths (447, 532, 635, and 808 nm), both individually and in combination, in human telomerase reverse transcriptase‐transformed retinal pigment epithelium (hTERT‐RPE) cells [32]. An increase in NO release from the cells was observed when exposed to all wavelengths, and either inhibitory or synergistic effects were produced by combinations of wavelengths, depending on which specific wavelengths were used [32]. However, no changes in NO levels were observed when succinate replaced pyruvate as the substrate and the cells were exposed to 635 nm light. Their findings led to the conclusion that the mechanism of light‐induced NO release from CCO may not be as simple as previously thought [32]. Furthermore, Lima et al. tested the effects of 660 nm irradiation in cells lacking CCO [83]. They showed that the increase in cell proliferation occurred independently of CCO, questioning the prevailing hypothesis that cell growth stimulation by PBM is promoted by CCO activation [83], indicating that other mechanisms might be involved, at least in this specific wavelength. Herrera et al. also reached similar conclusions, showing that the enhanced cell proliferation at 660 nm is promoted by different mitochondrial pathways other than by CCO activation, but rather by increased fatty acid oxidation [39]. Although Pope and Denton found that CCO activity was enhanced by 808 nm irradiation, they showed that the response does not obey power density reciprocity (where combining different power densities and exposure times resulting in the same light fluence should result in similar responses), indicating that this is probably not a simple photochemical effect [19].

In short, our analyses showed results that differ from what may be typically expected from PBM research with mitochondria, since there is no increase in OXPHOS or maximal respiratory capacity, as could be assumed from the CCO activation hypothesis. On the other hand, these results are aligned with the studies that challenge the CCO activation hypothesis [20, 39, 83], suggesting that: (1) the enhancement of mitochondrial metabolism may not have originated within the mitochondrial electron transport system, but probably is dependent on the mitochondrial network [85] and originated on different mitochondrial pathways [39, 83]; (2) it is possible that such effects regarding the increase in mitochondrial metabolism cannot be observed in real‐time, or immediately after irradiation at all, at least with the analysis methods employed here, as previous studies in the literature usually report the observation of late effects [39]; (3) CCO activity could potentially be enhanced in dysfunctional mitochondria, where elevated NO levels and decreased ATP production are frequently linked to disease‐related pathological conditions or deficiencies [1], and PBM would contribute to restoring mitochondria from these perturbed conditions to normal levels.

## 5 Conclusion

In this study, the main biological effects of 635 nm irradiation under the respiratory control conditions studied in mouse liver isolated mitochondria were related to membrane transitory uncoupling in real‐time analysis and to an increase in basal respiration in post‐irradiation analysis. The swelling profile post‐irradiation also reflected the opening of the mPTP. The results presented here contributed to the paradigm of how each type of study, sample, and irradiation parameter can lead to different results when investigating PBM action mechanisms and accentuating its complexity. The effects shown are intrinsically linked to the in vitro model of isolated mitochondria, which was chosen with the sole purpose of investigating the effects of irradiation directly on the mitochondrial machinery, without the interference of the cell structure. Performing an investigation with this experimental design provided new evidence that allowed us to imply that the prevailing hypothesis for mitochondrial metabolism enhancement by light may not be as well established as it is usually assumed, and further investigations are necessary to improve our understanding of this topic. To pursue the natural progression of this study, further studies shall focus on comparing these fundamental effects with outcomes induced by irradiation in more complex models, such as cells and biopsies, as well as investigating tissues from different organ sources. Additionally, future studies should also explore other wavelengths to better understand the spectral sensitivity of mitochondria.

## Author Contributions

Natasha F. Mezzacappo: conceptualization, methodology, validation, formal analysis, investigation, data curation, writing – original draft, writing – review and editing, visualization, project administration. Natalia M. Inada: conceptualization, methodology, validation, writing – original draft, writing – review and editing, visualization, supervision, project administration. Edilene S. Siqueira‐Santos: methodology, resources. José Dirceu Vollet‐Filho: writing – review and editing. Roger F. Castilho: conceptualization, resources, writing – review and editing, visualization. Michael L. Denton: writing – review and editing. Vanderlei S. Bagnato: conceptualization, methodology, resources, writing – review and editing, supervision, project administration, funding acquisition.

## Conflicts of Interest

The authors declare no conflicts of interest.

## Supporting information


**APPENDIX S1:**Supplementary information.

## Data Availability

The data that support the findings of this study are available from the corresponding author upon reasonable request.
