# Supplementary material for: Exploring Immediate Photon Effects From 635 nm Light on Mitochondrial Bioenergetics
Source: J Biophotonics. 2025 Jul 10;18(10):e202500162. doi: 10.1002/jbio.202500162 (PMC12507486; doi:10.1002/jbio.202500162)
Supplement: Supplementary file 1 — APPENDIX S1:Supplementary information. [file JBIO-18-e202500162-s001.docx]

**Supplementary material for:**

**Exploring immediate photon effects from 635 nm light on mitochondrial bioenergetics**

1. **Irradiation Characterization**

To characterize the irradiation, transmittance measurements of the Duran® glass cylindrical chamber and the mitochondrial sample were conducted, using a Varian Cary® 50 UV-Vis (Varian Medical Systems, EUA) spectrophotometer. We performed qualitative measurements regarding light delivery inside the respirometer using an isotropic probe optical fiber (IP85 model, Medlight, Swiss) connected to a USB4000 fiber optic spectrometer (OceanOptics®, EUA) (Figure S1A), with the SpectraSuite® (OceanOptics®, EUA) software. A power/energy meter LabMax TOP (COHERENT®, USA), sensor PM10 model was used to measure light outside the respirometer (Figure S1B). The measurements from the power/energy meter were transformed in irradiance according to Equation (1) below:

| $I= \frac{P}{A}$ | (1) |
| --- | --- |

where *I* is the irradiance, calculated in mW.cm^-2^, *P* is the power measured in mW with the power/energy meter and *A* is the detector area (2.89 cm^2^). The spectra from the SpectraSuite® software were transformed into irradiance measurements through a calibration using the measurements from the power/energy meter (Figure S2), rendered using Origin 2022 software (OriginLab®, USA).


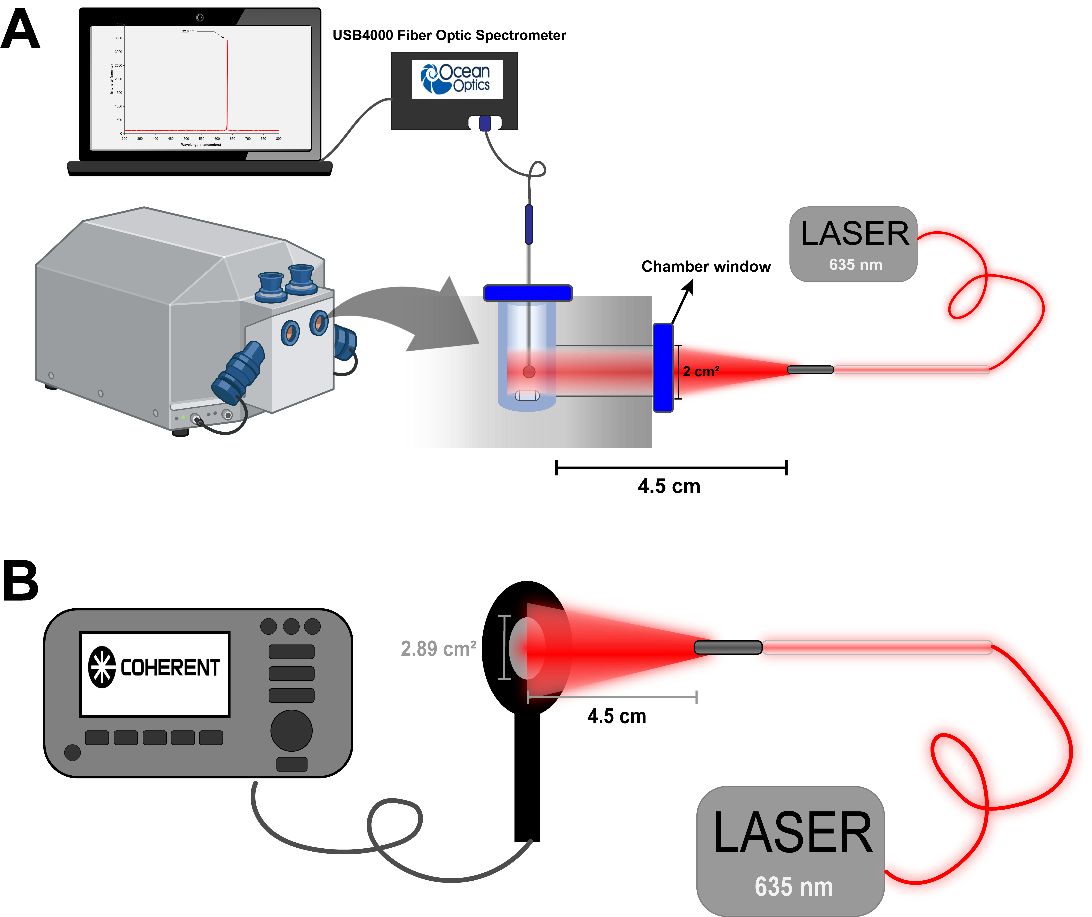


**Figure S1** - Experimental setup for characterization of irradiation delivered A) Inside the respirometer chamber; B) Outside the chamber (considering the same distance from the laser source to the chamber wall).


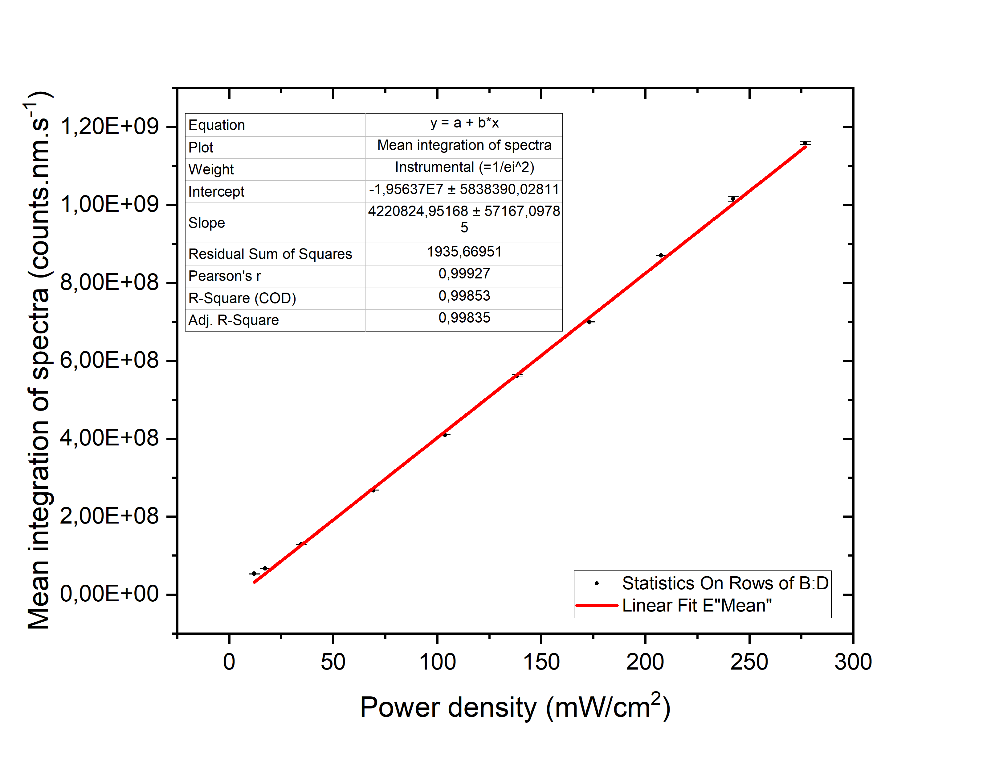


**Figure S2 -** Calibration curve generated from spectra integration and power densities measured outside the chamber.
